# Use of magnetic nanotrap particles in capturing *Yersinia pestis* virulence factors, nucleic acids and bacteria

**DOI:** 10.1186/s12951-021-00859-8

**Published:** 2021-06-21

**Authors:** Alexandra N. Ii, Shih-Chao Lin, Benjamin Lepene, Weidong Zhou, Kylene Kehn-Hall, Monique L. van Hoek

**Affiliations:** 1 School of Systems Biology, George Mason University, Manassas, VA 20110 USA; 2 Present Address: College of Life Sciences, National Taiwan Ocean University, 2 Pei-Ning Rd, Keelung, 202301 Taiwan; 3 Ceres Nanosciences, 9460 Innovation Drive, Manassas, VA 20110 USA; 4 Center for Applied Proteomics and Personalized Medicine, George Mason University, Manassas, VA 20110 USA; 5 Present Address: Department of Biomedical Sciences and Pathobiology, Virginia Polytechnic Institute and State University, Blacksburg, VA 24060 USA

**Keywords:** *Yersinia pestis*, DNA, Nucleic acid, Protein, Proteomics, Sample preparation, Plague, LcrV, F1 antigen, T3SS

## Abstract

**Background:**

Many pathogens, including *Yersinia pestis,* cannot be consistently and reliably cultured from blood. New approaches are needed to facilitate the detection of proteins, nucleic acid and microorganisms in whole blood samples to improve downstream assay performance. Detection of biomarkers in whole blood is difficult due to the presence of host proteins that obscure standard detection mechanisms. Nanotrap® particles are micron-sized hydrogel structures containing a dye molecule as the affinity bait and used to detect host biomarkers, viral nucleic acids and proteins as well as some bacterial markers. Nanotraps have been shown to bind and enrich a wide variety of biomarkers and viruses in clinically relevant matrices such as urine and plasma. Our objective was to characterize the binding ability of Nanotrap particle type CN3080 to *Y. pestis* bacteria*,* bacterial proteins and nucleic acids from whole human blood in order to potentially improve detection and diagnosis.

**Results:**

CN3080 Nanotraps bind tightly to *Yersinia* bacteria, even after washing, and we were able to visualize the co-localized Nanotraps and bacteria by electron microscopy. These magnetic hydrogel Nanotraps were able to bind *Yersinia* DNA, supporting the utility of Nanotraps for enhancing nucleic acid-based detection methods. Nanotraps were capable of increasing *Y. pestis* nucleic acid yield by fourfold from whole human blood compared to standard nucleic acid extraction. Interestingly, we found CN3080 Nanotraps to have a high affinity for multiple components of the *Yersinia* type III secretion system (T3SS), including chaperone proteins, Yop effector proteins and virulence factor protein LcrV (V). Using Nanotraps as a rapid upstream sample-prep tool, we were able to detect LcrV in human blood by western blotting with minimal blood interference in contrast to direct western blotting of blood samples in which LcrV was obscured. We were able to computationally model the interaction of LcrV with the CN3080 Nanotrap dye and found that it had a low delta-G, suggesting high affinity. Importantly, Nanotraps were also able to enhance detection of secreted *Yersinia* proteins by mass spectrometry.

**Conclusion:**

Upstream use of magnetic CN3080 Nanotrap particles may improve the downstream workflow though binding and enrichment of biomarkers and speed of processing. Utilization of Nanotrap particles can improve detection of *Yersinia pestis* proteins and nucleic acid from whole human blood and contribute to downstream assays and diagnostics including molecular methods such as sequencing and PCR and protein-based methods.

**Graphic Abstract:**

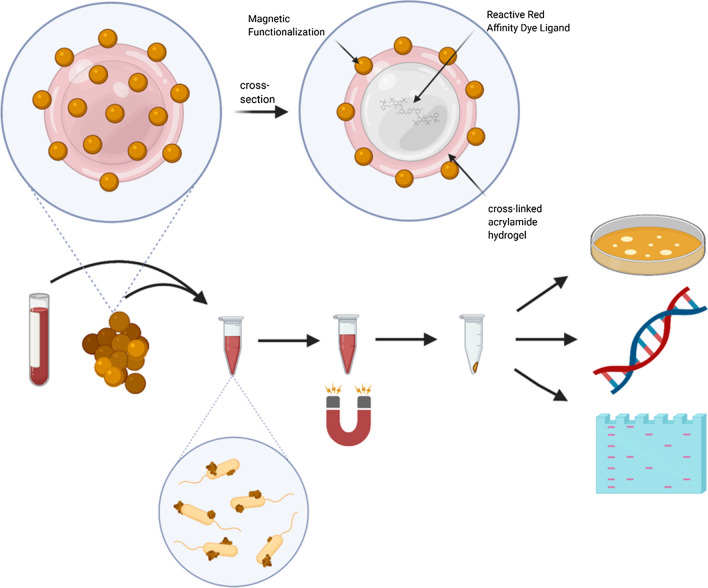

**Supplementary Information:**

The online version contains supplementary material available at 10.1186/s12951-021-00859-8.

## Introduction

*Yersinia (Y.) pestis* is the causative agent of plague and is considered a Category A biothreat agent by the National Institute of Allergy and Infectious Diseases and classified as a select agent by the Centers for Disease Control due to its infectivity for humans [1]. This organism was responsible for the Black Death plague that decimated the population of Europe in the fourteenth century. This gram-negative bacterium has a well-characterized pathogenicity for humans and other mammals via a vector-borne life cycle involving fleas and rodents. There continue to be active zoonotic outbreaks of plague worldwide, including in the western United States and Madagascar [2, 3]. Improved and less expensive methods of detection would contribute to treating these infections, and could be enabled by Nanotrap® particles.

When humans are infected with *Yersinia pestis*, bacteria can be found in the blood due to initial replication followed by severe septicemia [1, 4], lending an opportunity for blood-based diagnostics and detection. The gold standard for diagnosing bacterial infections including plague is by culture techniques from patient blood samples; however, culturing takes multiple days to obtain results and culture results from blood are variable [5]. Thus, improving the isolation and detection of *Y. pestis* from whole blood samples addresses a major clinically relevant need. In addition, new upstream sample preparation methods to bind, concentrate and protect *Y. pestis* biomarkers (such as nucleic acids or proteins) from patient blood samples may improve the sensitivity of downstream detection, such as PCR-based or antibody-based detection methods.

**Fig. 1:**
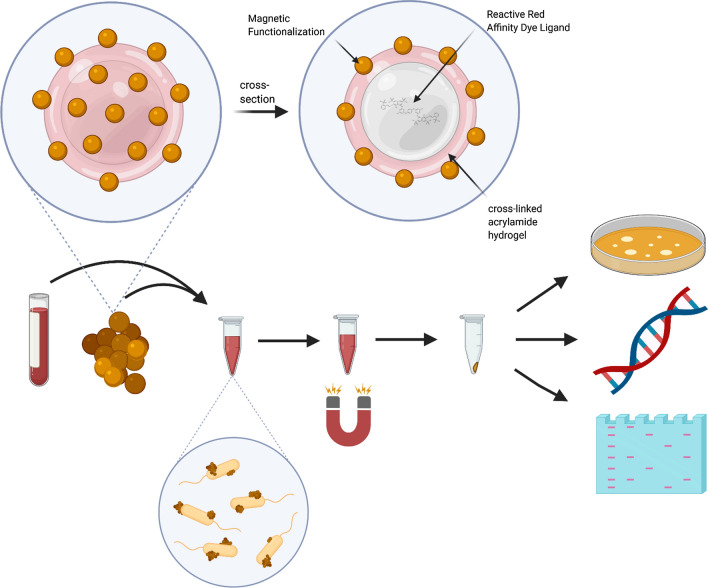
Nanotrap workflow. Magnetic Nanotraps (red spheres) can be added to challenging matrices, such as blood. Nanotraps adhere to microbial proteins and nucleic acid as well as bacteria. Nanotraps are pulled down using a magnet and interfering blood can be removed and used for further analysis. The magnetic pellet can be rapidly processed through downstream applications for diagnostic purposes

Nanotrap particles are micron-sized hydrogel structures made of cross-linked N-isopropylacrylamide-acrylic acid copolymers which have been used to detect host biomarkers, viral nucleic acids and proteins as well as some bacterial markers [6–13]. Nanotrap particles contain large, planar dye molecules as affinity bait encased inside the particle produced by co-polymerization of *N*-isopropylacrylamide through precipitation polymerization [14]. For this study, Reactive Red 120 dye is the incorporated bait molecule (see Fig. 1). These Nanotrap microparticles have been well characterized for their ability to bind to and enhance detection of biomarkers including human growth hormone and platelet derived growth factor [15, 16]. One interesting aspect of these hydrogel particles and the dye bait is the “non-specificity” of their binding; that is, unlike an antibody or a specific binding protein, we are able to use the broad binding capability of the dye bait to “sample” a wide range of proteins and other biomarkers that can bind to this bait. By altering the affinity dye bait inside the hydrogel particle, for example to Cibacron Blue, the binding profile of the particles shifts to a different spectrum of the available biomarkers [15]. This can enable the enhanced detection of low abundance biomarkers [14]. Thus, the particles can be used to sample proteins or nucleic acids from a wide variety of bacteria, viruses, cells or other biological samples. By introducing this sampling method to a complex biological matrix, such as whole blood, successful biomarker detection would have even greater clinical significance.

The CN3080 Nanotrap particles have been functionalized via decoration with magnetic microparticles (80–100 nm), enabling rapid sample pull-down, without the need for centrifugation, enabling possible future automation of the workflow. Other formulations of magnetic nanoparticles have previously been used for bacterial separation [17, 18], or bacterial protein binding though use of specific bait molecules [19], demonstrating the utility of this application.

We sought to apply this property to the detection of bacteria, proteins and also nucleic acids, to see what bacterial molecules could bind to these hydrogel particles. For example, if a *Yersinia pestis* protein of interest is bound to the Nanotrap hydrogel particles, it could be useful as an upstream step in a workflow used to detect the biothreat agent bacteria. If *Yersinia* DNA was bound to the Nanotrap, molecular diagnostic tools such as PCR can be used to specifically identify the organism. If this detection could be performed from a complex and challenging biological matrix, such as whole blood, it would have even greater clinical relevance.

These hydrogel microparticles have also been shown to bind viral nucleic acids, viral capsid and even intact viruses [6, 7, 10–12]. Recently, these particles have been functionalized via the incorporation of magnetic microparticles, enabling a much more rapid pull-down without the need for centrifugation and possibly enabling future automation of the workflow. The interactions of bacteria or bacterial biomarkers with Nanotraps have not been well-studied. Nanotraps have been shown to bind and enrich the outer membrane protein of *Borrelia burgdorferi*, the bacterium that causes lyme disease [9] and the lipoarabinomannan of *Mycobacterium tuberculosis* [20]. In this work, we characterize the ability of Nanotraps to interact with *Yersinia pestis* (pst-pgm-) bacteria, proteins and nucleic acids.

## Materials and methods

### Blood

Pooled, mix-gender de-identified whole human blood with K2EDTA (Catalog #: HUMANWBK2PZN) was obtained from BioIVT (www.bioivt.com).

### Bacteria

The BSL2 strain *Yersinia pestis* pgm-pst- is a derivative avirulent strain of *Yersinia pestis* CO92 and was obtained from Dr. Ramin Hakami with permission of USAMRIID (RG Panchal, USAMRIID) [21–23]. This strain is pigmentation (pgm)-deficient and cured of the plasminogen-activator-encoding pPst plasmid (thus pgm-pst-) and thus is avirulent for humans, and can be safely used at Biosafety level 2 [21–23].

Bacteria were streaked onto Brain Heart Infusion agar plates from a frozen (− 80 °C) glycerol stock and grown at 28 °C. A single colony was isolated and used to inoculate Brain Heart Infusion broth (BHI) and grown for 24–36 h at 28 °C with shaking at 180 rpm following published methods [24]. Cultures were enumerated using McFarland readings (Benchtop Densitometer Turbidimeter, McFarland Units, DEN-1, Grant Instruments) and verified by plating. Bacteria were pelleted and resuspended in Dulbecco’s Phosphate Buffered Saline (DPBS) to a McFarland value of 0.5 (a McFarland value of 0.5 is equivalent to 1 × 10^7^ CFU/mL for this organism).

### Nanotraps

Magnetized Nanotrap® CN3080 particles from Ceres Nanoscience (www.ceresnano.com) were used. These particles contain Reactive Red 120 Dye as the chemical bait. Nanotraps were provided from Ceres either as a sterile solution of particles at 5 mg/mL in buffer, or as a lyophilized pellet pre-aliquoted in a tube (0.25 mg Nanotraps). The estimated size of these particles is 400–900 nm decorated with 80–120 nm magnetic spheres. In order to separate magnetic Nanotraps from a solution, the GE MagRack 6 was used. The magnetic bar was placed next to the microcentrifuge tube for approximately 5 min in order to “pull-down” the particles.

### Bacterial binding to nanotraps

Bacterial binding to Nanotraps was determined by adding 1 × 10^5^ CFU bacteria to 200 µL blood. 50 µL (0.25 mg) of wet Nanotraps or 0.25 mg lyophilized Nanotraps were added to the sample and incubated for the indicated time at the indicated temperature, or for 30 min if no time is indicated. Following incubation, 800 µL DPBS was added to Nanotrap tubes and Nanotraps were pelleted using the magnetic bar. The supernatant was carefully removed and the pellet was washed with an additional 800 µL DPBS if specified. 100 µL DPBS was added to fully resuspend the Nanotrap pellet. 1:10 serial dilutions were conducted and 5 µL spot dilutions were plated (n = 5) on to Tryptic Soy Agar with 5% sheep’s blood or BHI agar plates to be enumerated after 36 h at 28 °C. Additionally, bacteria were plated with dilutions of Nanotraps in DPBS to verify a lack of growth inhibition.

### Bacterial stability with nanotraps

Bacterial stability was determined by adding 1 × 10^5^ CFU to whole human blood. 50 µL of wet Nanotraps (or 0.25 mg of lyophilized Nanotraps) were added to the sample and incubated for the indicated time at the indicated temperature, or for 30 min if no time is indicated. Following the incubation time, 10 µL of the thoroughly mixed suspension were diluted into 90 µL DPBS and 1:10 spot dilutions were conducted as stated.

### Nucleic acid preparation

Genomic DNA (gDNA) was prepared from samples using the DNeasy UltraClean Microbial kit (Qiagen) from *Yersinia* bacteria grown at 28 °C in BHI media. The quality of the gDNA was assessed by running on agarose gels. The quantity was determined by Nanodrop reading 260/280 nm ratio. These samples were used as positive controls for further experiments.

### Nucleic acid binding to nanotraps

Nanotrap samples were processed for plating experiments such as “bacteria incubated with Nanotraps”, and were then processed for nucleic acid extraction using the DNeasy UltraClean Microbial kit with the following modifications: DPBS was added 4:1 to blood Nanotrap samples. Samples were vortexed to reconstitute solidified blood and centrifuged following the kit protocol. Supernatant was removed. Washed samples were resuspended in the same volume of DPBS, centrifuged, and supernatant was removed. The particle pellet was resuspended in 300 µL PowerBead solution and Qiagen protocol was continued as instructed.

### Primer design and validation

*Yersinia* 16 s rRNA primers (Thermo Fisher Scientific) 5′-AGAGTTTGATCCTGGCTCAG-3′ and 5′-GGTTACCTTGTTACGACTT-3′ were identified in literature [25]. qPCR was performed using primers designed against *Yersinia* Ferric Uptake Regulator gene (Fur): 5′GATTGCGGCAAAGTGATCG-3′ and 5′-GCAATTGCCGGTTTCACAGT-3′. Validation of the primers was performed by serial dilutions of *Y. pestis* CO92 DNA obtained from BEI Resources (NR-2717, Manassas, VA), alongside extracted *Y. pestis* pgm-pst-gDNA, with appropriate negative controls.

### PCR detection of nucleic acids

Standard PCR was conducted using Q5 High-Fidelity 2 × Mastermix (New England Biolabs) using a thermalcycler (BioRad CFX96) with the following protocol: 98 °C for 30 s, followed by 25 cycles of 98 °C for 10 s, 60 °C for 30 s, and 72 °C for 30 m, followed by 72 °C for 2 min. The PCR product was run on a 1% agarose gel, stained using SYBR-Safe in TAE (Thermo Fisher Scientific), and visualized using the Bio-Rad ChemiDoc imager. qPCR was conducted using SYBR Green Perfecta Mastermix (Quantabio) with the following protocol: 95 °C for 60 s, with 40 cycles of 95 °C for 5 s, 52 °C for 15 s, and 68 °C for 10 s, with plate reads at the end of each cycle (BioRad CFX96). Quantitative values were obtained based on standard curve calculations through Bio-Rad CFX96 software manager.

### Bacterial lysate protein binding to nanotraps

A 24–36 h culture was grown at 28 °C or 37 °C on BHI agar or in BHI media. Plated cultures were scraped into 20 mM Tris HCl. Liquid cultures were centrifuged for 6000×*g* for 10 m, then the bacterial pellet was resuspended in cold 20 mM Tris HCl. Cells were sonicated on ice using the QSonica for 30 s at 40% amplitude, followed by 30 s pauses for 10 cycles, or until turbidity dissipated. Whole cell lysate (WCL) was centrifuged for 12,000×*g* for 15 m, then the supernatant was filtered using a 0.22 µm filter. Protein concentration was measured, then the cell lysate was aliquoted and stored at − 80 °C until further use. Liquid or lyophilized CN3080 Nanotraps were resuspended in 200 µL cell lysate at a known concentration, diluted in DPBS. Samples were incubated with mixing at room temperature for 30 m or 2 h, respectively. 800 µL DPBS was added prior to pelleting on a magnetic rack. Supernatant was discarded. If specified as washed, the CN3080 Nanotrap pellet was fully resuspended off the magnetic rack in 800 µL DPBS, pelleted, and supernatant was discarded.

### Secreted protein binding

The supernatant (BHI) of a 37 °C grown culture was removed following centrifugation and filtered through a 0.22 µm filter. Filtration product containing secreted proteins was diluted in DPBS or 1:4 whole human blood: DPBS. 1 mL of sample was added per tube of lyophilized particles. Additionally, larger volumes of culture supernatant were tested using the same ratio of 1 mL per tube of lyophilized particles (0.75 mg Nanotraps per 3 mL undiluted supernatant).

### Intact whole cell binding

A 24–36 h culture of *Y. pestis* was grown at 28 °C or 37 °C, centrifuged for 6000×*g* for 10 m and resuspended to 0.5 McFarland. 0.25 mg Nanotraps were resuspended in 1 × 10^7^ CFU/mL and mixed at room temperature for 2 h. 800uL DPBS was added prior to pelleting on magnetic rack. Supernatant was discarded.

### Mass spectrometry method

After pelleting on a magnetic rack and aspirating supernatant, Nanotrap samples were mixed with 20 μL of 8 M urea and incubated at 50 °C for 2 min. After centrifugation, the supernatant was reduced with 10 mM DTT, alkylated with 50 mM iodoacetamide, and digested with trypsin for 2 h at 37 °C. The digestion mixture was desalted with ZipTip, dried in SpeedVac, then reconstituted with 10 µL of 0.1% formic acid. LC–MS/MS experiments were performed on an Orbitrap Fusion (ThermoFisher Scientific, Waltham, MA, USA) equipped with a nanospray EASY-nLC 1200 HPLC system. Peptides were separated using a reversed-phase PepMap RSLC 75 μm i.d. × 15 cm long with 2 μm, C18 resin LC column. The mobile phase consisted of 0.1% aqueous formic acid (mobile phase A) and 0.1% formic acid in 80% acetonitrile (mobile phase B). After sample injection, the peptides were eluted by using a linear gradient from 5 to 40% B over 60 min and ramping to 100% B for an additional 2 min. The flow rate was set at 300 nL/min. The Orbitrap Fusion was operated in a data-dependent mode in which one full MS scan (60,000 resolving power) from 300 to 1500 Da using quadrupole isolation, was followed by MS/MS scans in which the most abundant molecular ions were dynamically selected by Top Speed, and fragmented by collision-induced dissociation (CID) using a normalized collision energy of 35%. “Peptide Monoisotopic Precursor Selection” and “Dynamic Exclusion” (10 s duration), were enabled, as was the charge state dependency so that only peptide precursors with charge states from +2 to +4 were selected and fragmented by CID. Tandem mass spectra were searched against the NCBI *Yersinia pestis* database (ASM906.1) using Proteome Discover version 2.1. Mass tolerance for precursor ions was 2 ppm, and mass tolerance for fragment ions was 0.5 Da. Data were analyzed with oxidation (+15.9949 Da) on methionine as a variable modifications, and carbamidomethyl cysteine (+57.0215) as a fixed modification. A 1% false discovery rate (FDR) was used as a cut-off value for reporting peptide spectrum matches (PSM) from the database.

### Relative enrichment value of *Yersinia* proteins bound to CN3080 nanotraps

The number of peptide spectra matches (PSMs) for each protein (including one hits) within a sample was summed, giving the total number of PSMs within a MS sample. For each protein, the individual number of PSMs was divided by the total number of PSMs within the sample. This process was conducted for all MS samples. The enrichment value was determined through normalization by dividing the number PSM/total PSM for a Nanotrap sample with the PSM/total PSM with its respective control. Relative Enrichment values comparing whole cell Nanotrap and lysate Nanotrap samples were also calculated. A PSM value of 5 in either the Nanotrap or control sample was set as the minimum to be considered for enrichment calculation.

### Western blot method

Following incubation, Nanotraps were pelleted on magnetic rack and supernatant was removed. Pellets were resuspended in 50 µL SDS sample buffer (ThermoFisher). Proteins were denatured and eluted from Nanotraps by heating at 90 °C for 10 m, pelleting on magnetic rack, and loading 15 µL onto a 4–12% Bis Tris Protein gel. Samples were run at 200 V for 32 min, transferred onto PVDF membrane, and blocked in TSB-T 5% nonfat milk solution for 1 h. Membranes were probed overnight at 4 °C with a 1:50 LcrV (V-antigen, BEI NR-3831) or 1:50 F1 antigen antibody (Invitrogen MA1-7427) in TSB-T nonfat milk following the manufacturers suggestion. Secondary probing was conducted with 1:1000 anti-goat (R&D HAF017) or goat anti-mouse antibodies (Invitrogen #31,430) and bands were visualized using West Femto chemiluminescent solution and imaging on a ChemiDoc instrument (Bio-Rad).

### Scanning electron microscopy method

Standard methods were used to prepare and visualize bacterial samples [26]. Briefly, bacteria were grown at 28 °C for 24–36 h in BHI, centrifuged at 6000×*g* for 10 m, and resuspended to 0.5 McFarland in DPBS. 1 mL of resuspension was bound to lyophilized CN3080 Nanotraps following standard unlysed bacteria binding protocol, with variations of bacterial concentrations. Samples were fixed for 30 min with a 5% glutaraldehyde solution in 0.1 M phosphate buffer (pH 7.2). Fixative was discarded and the Nanotrap pellet was washed twice with 0.1 M phosphate buffer. Following carbon coating, fixed cells and Nanotraps were visualized using the Hitachi SU-70 Schottky field emission gun scanning electron microscope at University of Maryland’s Advanced Imaging & Microscopy Laboratory (College Park, MD).

### Protein docking prediction

The protein structures of *Y. pestis* virulence factors PsaA and LcrV were directly downloaded from PDB (entries: 4F8P, 4JBU) and the chemical structure of Reactive Red 120 was retrieved from ChemSpider (http://www.chemspider.com). Structures were subsequently submitted to SwissDock for evaluating potential docking pockets by Native Binding Mode (NBM). The docking results were visualized with UCSF Chimera v1.13 in the space-filling model presenting the electrostatic potential surface according to Coulomb’s law. The lowest free energy predicted by SwissDock would be considered as the strongest binding interaction between Reactive Red 120 and PsaA or LcrV (Fig. 6a, b).

### Statistics and data analysis

Comparisons between Nanotrap and control samples were conducted using unpaired Student’s T-tests using GraphPad Prism software. Statistical significance was set at P < 0.05. Gene ontology was analyzed by PANTHER Overrepresentation Test (Released 20190711, GO Ontology database Released 2019-12-09) analyzed against the *Yersinia pestis* reference list.

### Availability of data and materials

The datasets supporting the conclusions of this article are included within the article and its additional files.

## Results

As part of a larger project to characterize the capabilities of a panel of Nanotraps, we previously screened a panel of available Nanotrap particles (Ceres Nanoscience) made with various dye cores, in both magnetic and non-magnetic formulations in order to identify a particle formulation that bound well to both bacterial and viral markers [12]. The formulation of Nanotrap (Particle type CN3080) that was chosen for further characterization was a magnetic version of the Nanotrap particle containing Reactive Red 120 dye. This particle type was selected for its ability to bind both to viral markers [12] as well as bacterial proteins. Here, we characterize the ability of CN3080 Nanotrap particles to interact with *Yersinia pestis* (pst-pgm-) bacteria, its proteins and nucleic acids.

The workflow for using Nanotrap particles is illustrated in Fig. 1. These hydrogel microparticles are magnetized to provide easy and rapid particle separation without centrifugation. The particles are added to the complex biological matrix such as blood or plasma and allowed to bind between 30 min and 2 h with rotation. The particles are then pelleted by magnetic pull-down, allowing for simplified target separation from technically challenging matrices like blood. Target products, such as viable bacteria, nucleic acid, and protein, can be eluted from the particles for downstream processing.

### CN3080 Nanotraps bind *Yersinia* bacteria visualized by electron microscopy

**Fig. 2:**
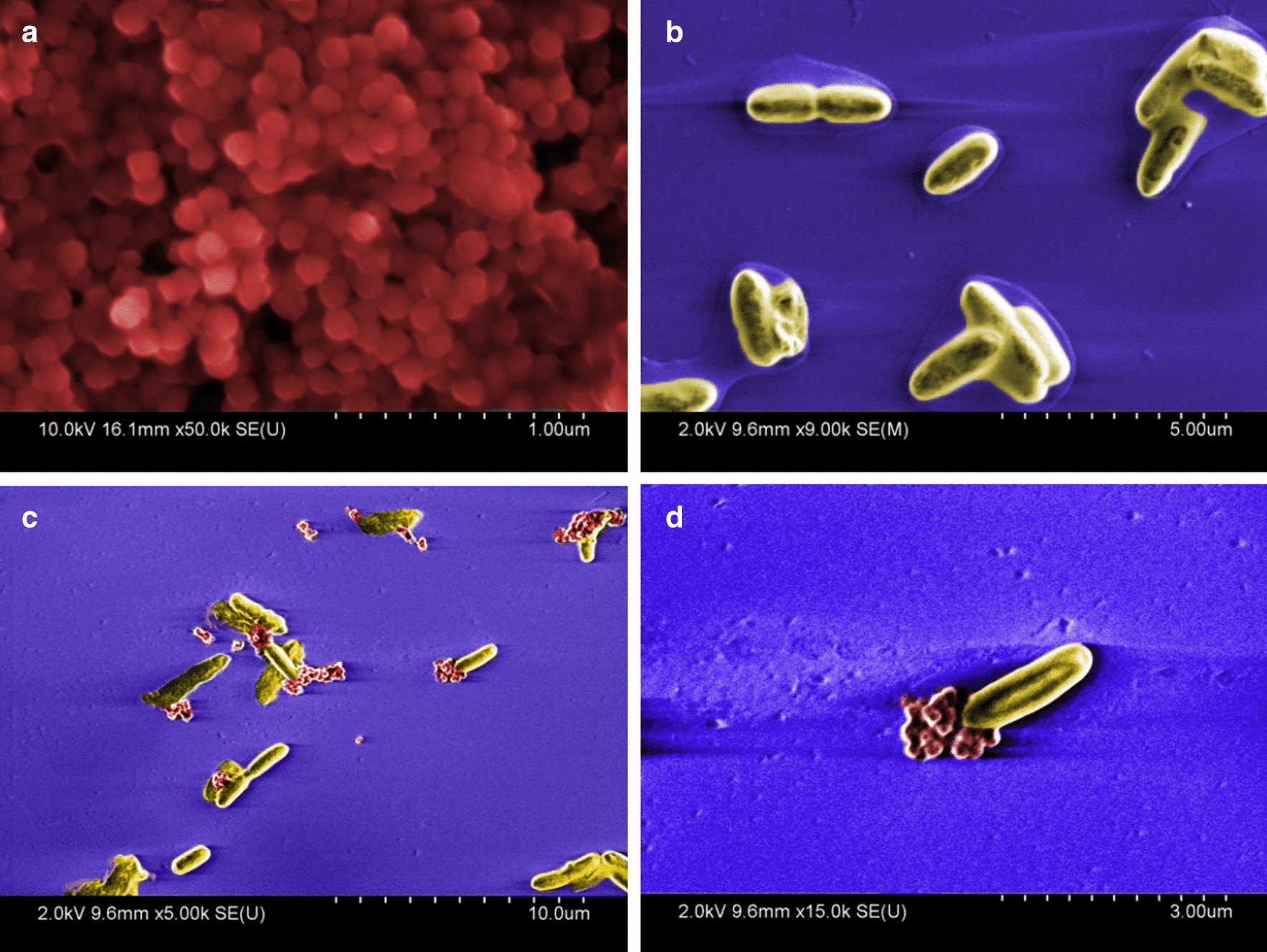
Scanning electron microscopy images of *Yersinia pestis* bacteria associated with CN3080 Nanotraps. **a** CN3080 Nanotraps (1 µm scale) without bacteria), **b** *Yersinia* bacteria without Nanotraps (5 µm scale), **c**, **d** *Yersinia* fixed following 2 h incubation with Nanotraps (10 and 3 µm scales)

We were able to observe the bacteria with Nanotrap particles by electron microscopy. The CN3080 Nanotraps are estimated to be 400–800 nm with magnetic spheres of approximately 80–120 nm in size decorating the surface, which can be seen in the figure (Fig. 2a). *Yersinia pestis* bacteria is estimated to be 1–3 µm long and 0.5–0.8 µm wide (Fig. 2b). As seen in panels 2C and 2D (Fig. 2c, d), the bacteria and Nanotraps appear to associate with each other on the surface of the bacteria when incubated together in PBS buffer.

### CN3080 Nanotraps bind *Yersinia* bacteria from whole human blood

**Fig. 3:**
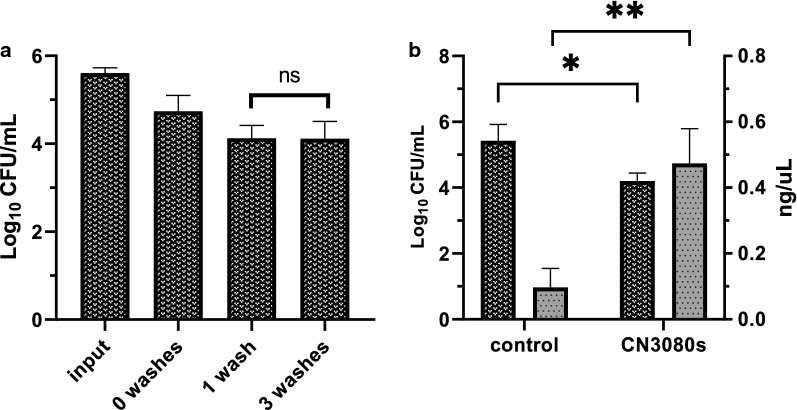
CN3080 Nanotraps bind *Yersinia* bacteria and enhance nucleic acid yield. **a** Following bacterial binding to CN3080s, bacteria were pulled down by magnetic rack and plated. The magnetic pellet was washed 1 or 3 times and plated to calculated CFU/mL. No significant decrease in CFU/mL was determined with repeated washes. **b** Following overnight incubation at 40 °C with CN3080s, a significant (P < 0.05) decrease in CFU was found relative to without CN3080s. Nucleic acid yield was significantly improved (P < 0.005) with use of CN3080s

We found that the *Y. pestis* bacteria associate tightly with the Nanotrap particles in whole human blood, even after washing (Fig. 3a). In this experiment, 7 × 10^4^ CFU were found associated with the Nanotraps after the first pull-down, and following subsequent washing steps, 1 × 10^4^ CFU were found to be still attached to the particles. No significant inhibition of bacterial growth was found with increased concentrations of Nanotraps up to 100uL/mL (Additional file 1: Figure S1).

CN3080 Nanotrap particles have an affinity for *Yersinia pestis* bacteria in undiluted whole human blood. Binding experiments show consistent bacterial recovery following 2 PBS washes. However, the addition of the Nanotrap particles to blood containing bacteria did not appear to increase stability of bacteria at elevated temperatures (Additional file 1: Figure S2, S3). Though Nanotraps were capable of binding and pulling bacteria out of solution, the rate of CFU decline was not improved, thus we conclude that the Nanotraps do not “stabilize” the bacteria.

Although Nanotrap particles do not enhance bacterial stability over time and at elevated temperatures, they do associate tightly with bacteria. In the experiment shown in Fig. 3b, 1 × 10^5^ CFU were spiked into 200 µL whole human blood and incubated at 40 °C for 24 h to promote release of nucleic acid while allowing some cell survival (Fig. 3b). CN3080 Nanotraps were pelleted from blood by centrifugation at 6000×*g*, resuspended, and plated. Centrifugation of the samples allowed detection of unbound bacteria. Control and Nanotrap samples were plated following incubation to determine the impact of Nanotraps and/or elevated temperature on bacterial survival. We found a significant decrease (20%) in viable bacteria (P < 0.05) when incubated at 40 °C for 24 h, indicating that Nanotraps do not stabilize *Yersinia pestis* against this elevated temperature.

### CN3080 Nanotraps facilitate nucleic acid extraction

We have been able to demonstrate binding of *Yersinia pestis* (pgm-pst-) genomic DNA from whole human blood spiked with 20 ng bacterial gDNA (Additional file 1: Figure S4). CN3080 Nanotraps were capable of enhancing detection of *Y. pestis* gDNA from whole human blood stored at 40 °C for 48 h. Though this does not establish increased temperature stability of *Yersinia* gDNA with the addition of Nanotraps, it does suggest that Nanotraps are capable of associating with bacterial nucleic acid and improving overall yield. The same CN3080 Nanotraps have been shown to bind Venezuelan Equine Encephalitis Virus nucleic acid in blood [12].

In the same experiment, shown in Fig. 3b, 1 × 10^5^ CFU were spiked into 200 µL whole human blood and incubated at 40 °C for 24 h to promote release of nucleic acid from the death of some bacteria at this elevated temperature. CN3080 Nanotraps were pelleted from blood by centrifugation at 6000×*g*, resuspended (and plated, see above), then nucleic acid was extracted from the remaining sample. Despite a significant decrease in viable bacteria (P < 0.05), we found significant improvement in nucleic acid yield using CN3080 Nanotraps (P < 0.005). These data further support the utility of Nanotraps for enhancing DNA detection.

### Nanotraps enhance detection of virulence factors by Mass-spectrometry

**Fig. 4:**
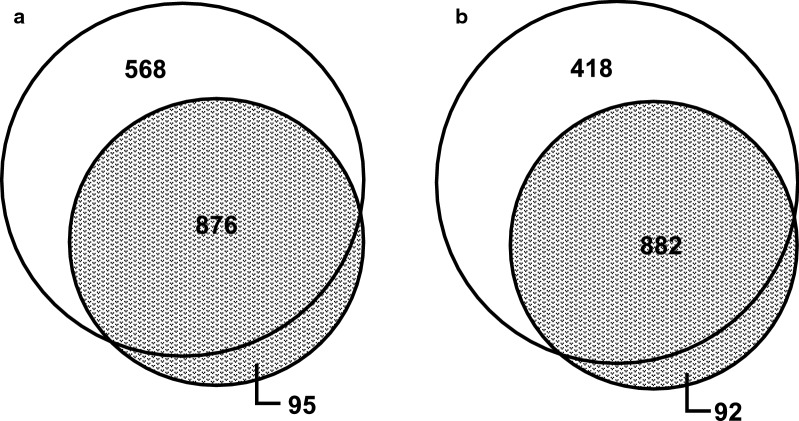
CN3080 Nanotraps bind both 28 °C and 37 °C expressed *Yersinia* proteins. Venn diagrams of *Yersinia* proteins found by mass spectrometry. **a** Total proteins found from 28 °C grown *Yersinia* control samples (light grey) and proteins found bound to CN3080s (dots) following incubation with 28 °C grown bacterial proteins. **b** Total proteins found from 37 °C grown controls (dark grey) and proteins found bound to CN3080s (dots) following incubation with 37 °C grown proteins

We conducted experiments to assess the ability of Nanotraps to bind and enrich *Y. pestis* proteins under a variety of growth conditions, including varied temperatures and media (Table 1, Samples 1–6 described). Additionally, from 37 °C grown *Y. pestis,* we examined the affinity of Nanotraps for intra- and extracellular proteins by binding to both un-lysed cells and whole cell lysate (WCL). We found that Nanotrap particles are capable of enhancing detection of *Yersinia* proteins from both 28 °C and 37 °C growth conditions, reflecting the temperature of both environmental and clinical samples respectively (Fig. 4). The use of CN3080 Nanotraps resulted in successful recovery of approximately 70–90% proteins found in respective whole-cell lysate (control) samples (Additional files 2, 3, 4, 5, 6, 7: Tables S1–S6). Within Sample S1 (28 °C grown whole cell lysate incubated with Nanotraps), we found that approximately 70% of the total proteins detected in whole cell lysate were identified on Nanotraps (Fig. 4a), similarly for the bacteria grown at 37 °C (Fig. 4b). For 258 of these proteins, we found a concentrating effect where the number of identified peptides (as #PSM) was greater within the Nanotrap sample (Additional Tables). However, no significant relationship was found between these proteins (Additional file 1: Figure S6). Although CN3080 Nanotraps did not recover all proteins found in the whole cell lysate samples, we found approximately 60% of these proteins present in other CN3080 samples, suggesting that the results are not due to a lack of affinity and occur due to sample variability.

**Table 1:** Sample identification key for Fig. 7*Yersinia pestis* samples submitted for mass spectrometry

Sample name	Sample description
W28A	WCL of 28 °C agar plate grown *Y. pestis* bound to 0.25 mg CN3080s
W37A	WCL of 37 °C agar plate grown *Y. pestis* bound to 0.25 mg CN3080s
W37B,	WCL of 37 °C broth grown *Y. pestis* bound to 0.25 mg CN3080s
W37BW	WCL of 37 °C broth grown *Y. pestis* bound to 0.25 mg CN3080s, washed with DPBS
U37	Unlysed 37 °C broth grown *Y. pestis* bound to 0.25 mg CN3080s
S37	*Y. pestis* Supernatant (secreted proteins) bound to 0.25 mg CN3080s

*Yersinia* genes on the pCD1 plasmid, specifically those encoding the type III secretion system are known to be upregulated at 37 °C, while low levels of these proteins are produced at 28 °C [27]. Within our 28 °C sample set, we identified many of these proteins with greater numbers of peptide spectra counts compared those found in the respective control sample (Y0075, ParD, LcrG, YopN, YscE, YscM, Caf1A). Specifically, LcrG, YPMT1.69, YPMT1.71, and YPMT1.68A could not be found in 28 °C samples without CN3080 Nanotraps. This result is representative of the Nanotrap’s ability to concentrate low-level proteins and allow detection by mass spectrometry.

When sorted by the number of PSMs, the top 37 °C expressed proteins pulled down by CN3080 Nanotraps are predominantly enzymes and chaperone proteins (Table 2, Additional file 3: Tables S2, Additional file 4: Table S3, Additional file 5: Table S4). Within the top 20 proteins found in CN3080 Nanotrap samples, 20% of proteins are involved in protein folding, including isomerase FkpA and chaperone proteins Tig, DnaK, GroL, and ClpB. The top 20 most abundant proteins found in Nanotrap samples can also be found within the top 10% of respective controls, indicating protein abundance plays a role in CN3080 Nanotrap binding. However, some frequently occurring proteins possess higher numbers of PSMs in Nanotrap samples compared to control samples; these proteins include KatG, DnaK and Acs, which are consistently found enriched in whole cell lysate of 37 °C grown whole-cell lysate samples of *Y. pestis* bound to CN3080s (From Table 1, Samples W37A (agar-grown), W37B, and W37BW (broth-grown with Nanotraps, with and without washing), suggesting protein structure can influence binding and enrichment level.

**Table 2:** Top 5 proteins bound to CN3080 Nanotraps found by Mass Spectrometry found in 37 °C-grown *Yersinia pestis* samples (Whole Cell Lysate samples W37A, W37B, and W37BW (agar grown, broth-brown and broth-grown and washed Nanotraps, respectively), and unlysed bacteria Sample 5)

Entry	Gene names	Mass (kDa)	Gene ontology (biological process)	Gene ontology (cellular component)	Gene ontology (molecular function)
Q8ZIY3	groL	57.4	'De novo' protein folding; protein refolding	GroEL-GroES complex	ATP binding; unfolded protein binding
Q8ZJB2	tufA	43.2	Translational elongation	Cytoplasm	GTPase activity; GTP binding; translation elongation factor activity
Q9X6B0	katG	81.4	Cellular response to hydrogen peroxide; hydrogen peroxide catabolic process	Cytosol; periplasmic space	Catalase activity; heme binding; metal ion binding
Q8ZAN8	tufB	43.2	Translational elongation	Cytoplasm	GTPase activity; GTP binding; translation elongation factor activity
Q8ZIM7	dnaK	69.0	Cellular response to heat; cellular response to unfolded protein; chaperone cofactor-dependent protein refolding; protein refolding; response to unfolded protein	Cytoplasm; cytosol	ATPase activity; ATPase activity, coupled; ATP binding; heat shock protein binding; misfolded protein binding; protein folding chaperone; unfolded protein binding

To determine if CN3080 Nanotraps could concentrate *Yersinia pestis* proteins from dilute samples, bacterial supernatant containing secreted proteins was incubated with CN3080 Nanotraps overnight at 4 °C. We found that the addition of CN3080 Nanotraps enabled detection of secreted and membrane proteins by mass spectrometry (Table 3, Additional file 7: Table S6). This allowed detection of proteins from a low concentration supernatant sample without use of dialysis or standard protein concentrators. Proteins in this sample included virulence markers YopM, LcrV, and F1-antigen (caf1). In laboratory diagnosis, the F1 antigen is commonly used as a target for immunological-tests to detect *Y. pestis* [1]. thus, this result suggest that Nanotraps could potentially enhance the detection of F1 antigen in those tests. LcrV is a well-studied, secreted protein of *Y. pestis* [28, 29]. We were able to detect Nanotrap-associated LcrV through mass-spectrometry proteomics analysis. The spectra from that analysis are shown in Additional file 1: Figure S7, confirming the identification of this protein in association with CN3080 Nanotraps.

**Table 3:** Membrane and secreted proteins concentrated from 37 °C *Yersinia* supernatant by CN3080 Nanotraps.  All listed proteins were undetected by MS without CN3080s

Entry	Gene names (primary)	Gene ontology (biological process)	Gene ontology (cellular component)	Gene ontology (molecular function)
P17778	yopM	–	Cell outer membrane; extracellular region	Metal ion binding
Q0WG31	gptB	Phosphoenol-pyruvate-dependent sugar phosphor-transferase system	Cytoplasm; integral component of membrane	d-glucosamine PTS permease activity; protein-N(PI)-phosphohistidine-sugar phosphotransferase activity
Q7CGH2	hasF		Efflux pump complex; outer membrane	Efflux transmembrane transporter activity; porin activity
P0C7U7	lcrV*	Pathogenesis	Extracellular region	–
Q8ZC05	Gpt	GMP salvage; IMP salvage; XMP salvage	Cytosol; plasma membrane	Hypoxanthine phosphoribosyltrans-ferase activity; magnesium ion binding; xanthine phosphoribosyltrans-ferase activity
P26948	caf1*		Capsule; extracellular region	
Q8ZG77	ompA	Ion transport	Cell outer membrane; pore complex	porin activity
Q8ZH58	bamA	Gram-negative-bacterium-type cell outer membrane assembly; protein insertion into membrane	Bam protein complex; integral component of membrane	–
Q8ZBN2	eno	Glycolytic process	Cell surface; extracellular region; phosphopyruvate hydratase complex	Magnesium ion binding; phosphopyruvate hydratase activity
P31493	yopE	Negative regulation of phagocytosis; pathogenesis	Cell outer membrane	GTPase activator activity

* Proteins were chosen for computational modeling with dye, along with PsaA

Relative enrichment scores for proteins identified by mass-spectrometry were calculated by taking a samples’ normalized PSMs divided by normalized PSMs of its respective control. This score is indicative of the change in individual protein concentration in the sample. Proteins with enrichment scores ≥ 2.0 in 37 °C grown whole cell lysate (WCL) samples were assessed by gene ontology (Panther) and showed clustering based on biological process and molecular function (Additional file 8: Table S7). All established protein stabilization proteins were found within this data set including chaperone protein SurA and small heat shock proteins IbpA and IbpB (P < 0.005). Additionally, these proteins often exhibited oxioreductase, activity, ligase activity, and cyclic or heterocyclic compound binding (P < 0.0005). Similarly, within other 37 °C samples analyzed by mass spectrometry, we found additional proteins with similar biological processes. Within top enrichment scores of Sample W37BW (37 °C Broth-grown *Y. pestis* whole cell lysate with washed Nanotraps, Table 1), we identified a significant cluster of protein folding proteins such as chaperone proteins ClpB, DnaK, HtpG, along with trigger factor Tig and enzyme FkpA (P < 0.0005), alongside the protein stabilization proteins found on Nanotraps incubated with the whole-cell lysate of 37 °C agar-grown *Y. pestis* sample (Sample W37A, Table 1).

### CN3080 Nanotraps improve western blotting of LcrV protein in human blood

**Fig. 5:**
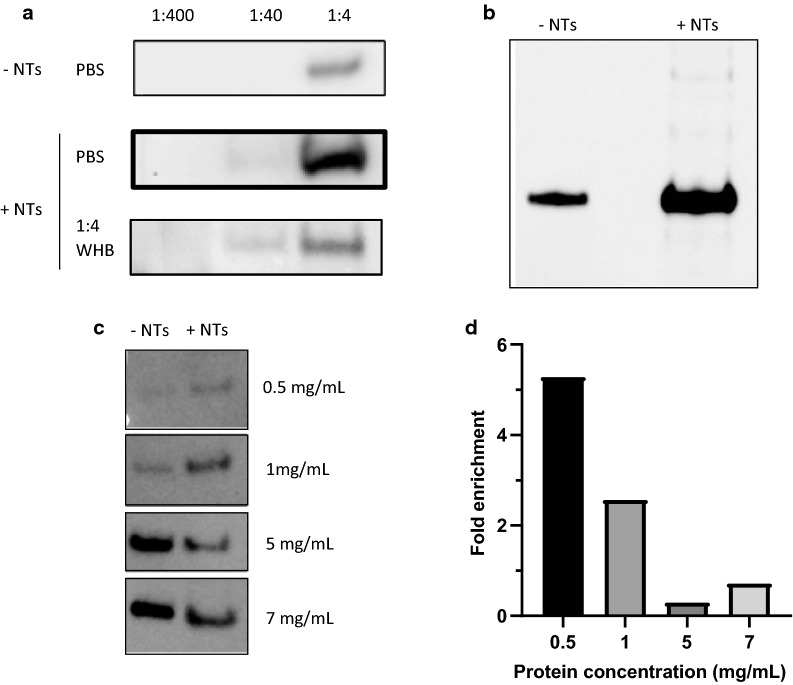
CN3080 Nanotraps bind *Yersinia* V-antigen in whole human blood. **a** V-antigen western blot of 1:4, 1:40, 1:400 dilutions of *Yersinia* supernatant in diluted human blood or PBS without (top) and with CN3080s (middle, bottom). **b** CN3080s effectively concentrate V-antigen from bacterial supernatant. **c**, **d** Fold enrichment of protein concentration is greater at lower initial protein concentrations

Given that both F1 and LcrV protein were detected in our mass spectrometry analysis, experiments were performed to confirm these results through western blot analysis. The F1 protein is a secreted 15 kDa protein of *Yersinia pestis* with a well-known role in inhibition of phagocytosis [30]. The LcrV protein is a secreted 37 kDa protein of *Yersinia pestis* that acts as a modulator of host cytokine production and regulator of effector protein translocation [28, 29]. Though F1 protein was observed by mass spectrometry in Nanotrap samples, it was not found to be enriched through western blotting (Additional file 1: Figure S5). Following 1:4, 1:40, and 1:400 dilutions of bacterial supernatant in DPBS or 1:4 diluted whole human blood, 1 mL samples were incubated with CN3080 Nanotraps for 2 h at 4 °C. LcrV (V-antigen) was detected by mass spectrometry (Additional file 1: Figure S7) and Western-blot bands could be detected at a greater intensity in the Nanotrap sample than in the corresponding control samples (Fig. 5a). We were unable to detect protein at the 1:40 dilution without use of Nanotraps. However, the limit of detection was reached even with Nanotraps prior to the 1:400 dilution. Following CN3080 Nanotrap overnight incubation at 4 °C with undiluted bacterial secreted protein, we were successfully able to concentrate LcrV (Fig. 5b). LcrV exists at low levels in the cell prior to bacterial secretion and T3SS assembly. Using 0.5, 1.0, 5.0, and 7.0 mg/mL *Yersinia* WCL, we were able to detect LcrV at our lowest concentration utilizing Nanotraps. The use of Nanotraps resulted in a 2.5 fold increase in band intensity from 1 mg/mL WCL and a fivefold increase from 0.5 mg/mL (Fig. 5c, d). However, at higher concentrations of V-antigen, the utility of Nanotraps decreased, consistent with prior reports. These data indicate that CN3080 Nanotrap enables the detection of V-antigen in blood and in bacterial lysates when it is present at low levels.

### CN3080 Reactive Red 120 dye binds virulence factors LcrV and PsaA

In order to gain insight on how CN3080 Nanotrap may interact with *Yersinia pestis* proteins, we performed computational protein docking of *Yersinia pestis* proteins against the Reactive Red 120 dye which is in the CN3080 Nanotrap particles. We selected LcrV and pH 6 antigen (PsaA) proteins given their elevated enrichment values found by mass spectrometry and the high degree of association with Nanotraps as visualized by western blotting.

**Fig. 6:**
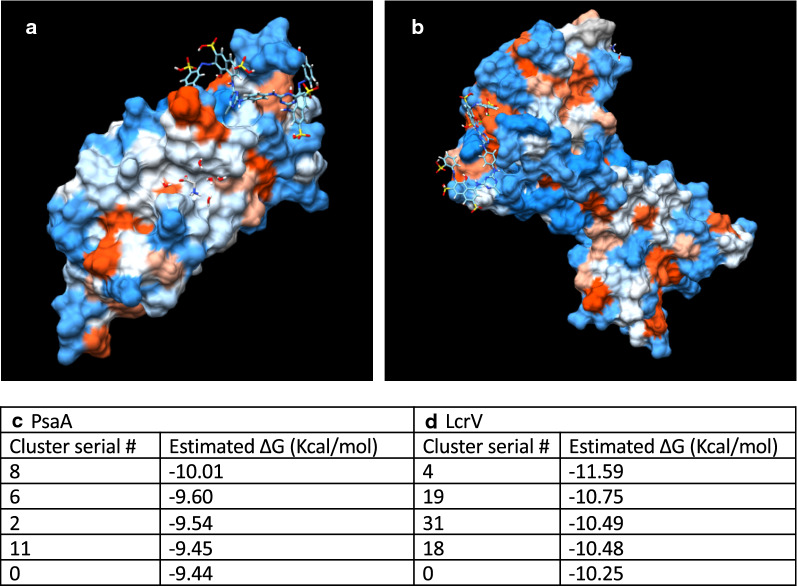
Modeling of interaction between Reactive Red-120 Dye and *Yersinia* proteins. Predictive modeling show multiple sites of strong interaction for Reactive Red (RR)-120 Dye with *Yersinia pestis* proteins PsaA and LcrV. **a** PsaA, **b** LcrV, **c** Estimated ΔG (Kcal/mol) for top PsaA-RR120 dye interactions. **d** Estimated ΔG (Kcal/mol) for top LcrV-RR120 dye interactions

PsaA is a 17 kDa protein known for its role as a fibrillar component expressed following engulfment by macrophages. It is exclusively expressed at 37 °C and acts as an obligate virulence factor. PsaA directly interacts with the human TRAF-type zinc finger domain-containing protein 1, and functions as an inhibitor of phagocytosis and promoter of host cell adherence. Because we consistently found this protein with elevated enrichment scores (2.80, 1.16, 2.11) within our 37 °C sample, we sought to visualize docking through modeling of this protein’s interaction with Reactive Red 120, the dye in CN3080 particles (Fig. 6a). SwissDock modeling was performed using the PDB structures for the proteins and the dye to predict the sites of interaction of this protein with the dye included in the CN3080 Nanotrap. The strongest ΔG value was − 10.0072 (Fig. 6c), suggesting a high level of affinity between this protein and Reactive Red 120. Multiple docking predictions were reported, with the majority of those found to be located near the C-terminal, suggesting that there may be multiple opportunities for this protein to be bound by the Reactive Red 120 dye molecule. This may explain the high levels of Nanotrap enrichment of this protein observed in our study.

As mentioned above, LcrV is a multifunctional virulence factor known for its roles in modulation of immune response and regulation of effector protein secretion in *Yesinia pestis*. LcrV expression is induced by temperature shift to 37 °C [27]. It is highly expressed in the 37 °C samples and is required for full virulence. LcrV is enriched in the 37 °C agar-grown whole bacterial lysate + Nanotrap samples by both mass spectrometry and western blotting. Based on our Swiss-Dock modeling of the interaction between LcrV and Reactive Red 120 (Fig. 6b), the dye in these Nanotraps, it has a very strong interaction with a ΔG value of 11.59 (Fig. 6d), supporting our experimental findings of enrichment of LcrV protein by using Nanotraps.

In further analysis, the active sites of PsaA and LcrV were highlighted; one for galactose-binding site (PsaA) [31] and the other for TLR2 interaction region (LcrV) [32] (Additional file 1: Figure S8A, B). The strongest binding sites of Reactive Red 120 were exactly correlated to these active sites. Also, the major interactions were shown to be hydrogen bonds (Additional file 1: Figure S8C, D, E), between active sites of LcrV and Reactive Red 120. In Figures S8A and S8B, these sites were labeled with distances of H-bonds which were plotted next to the highlighted graph.

Thus, the computational modeling supports the strong interaction of LcrV with the Reactive Red dye which is a component of the CN3080 Nanotrap, and thus may in part explain the enrichment of LcrV observed with the CN3080 Nanotrap observed in our experiments.

### CN3080 Nanotraps bind *Yersinia pestis* virulence factors

**Fig. 7:**
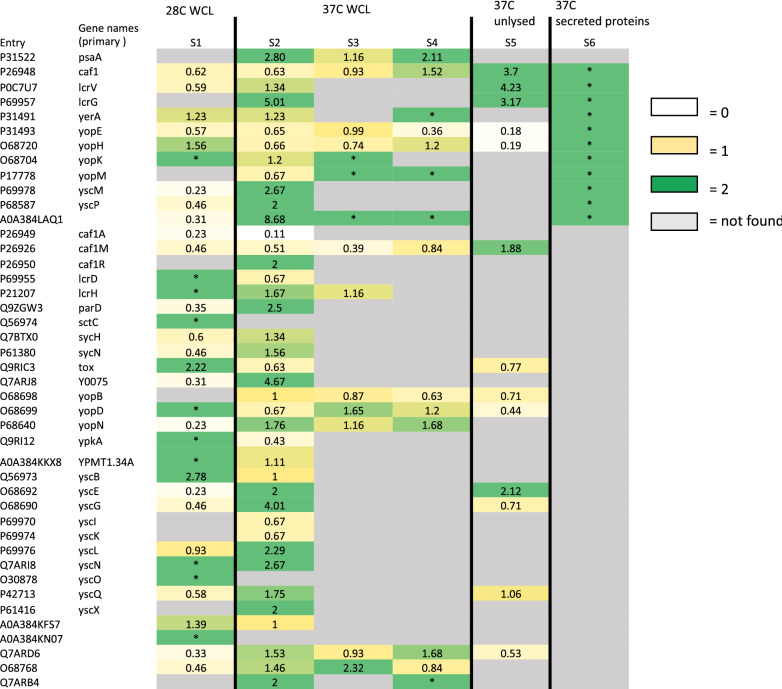
Virulence factor enrichment on Nanotrap. Virulence factors including proteins encoded on the pCD1 plasmid color coded by level of enrichment found within each MS sample. Proteins that were not found within the no Nanotrap control sample, but found within the Nanotrap sample are noted with asterisks (*). Boxes are colored according to enrichment value as shown in legend, grey = not found by Nanotraps)

We have shown that the Nanotrap particles enhance the mass-spectrometry based detection of multiple *Yersinia* virulence factors, including the well-known protein antigens F1 and LcrV (Fig. 7). Surface proteins such as F1 antigen (caf1) and F1 chaperone protein Caf1M showed higher levels of enrichment on CN3080 Nanotraps within whole bacteria bound to CN3080 Nanotraps, (Sample 5, see sample key in Table 1), relative to 37 °C whole cell lysate (WCL) samples (Samples 2, 3 and 4, Table 1). This may indicate more effective surface protein binding when Nanotraps are exposed to whole cells as opposed to entire lysed cell contents. Conversely, LcrG showed high affinity (~ fivefold) to Nanotraps in WCL lysate from agar-grown *Y. pestis* grown at 37 °C (Sample 2), but was not found on Nanotraps incubated with WCL lysate of *Y. pestis* grown at 28 °C (Sample 1) or in broth-grown bacterial cells grown at 37 °C (Samples 3, 4).

**Fig. 8:**
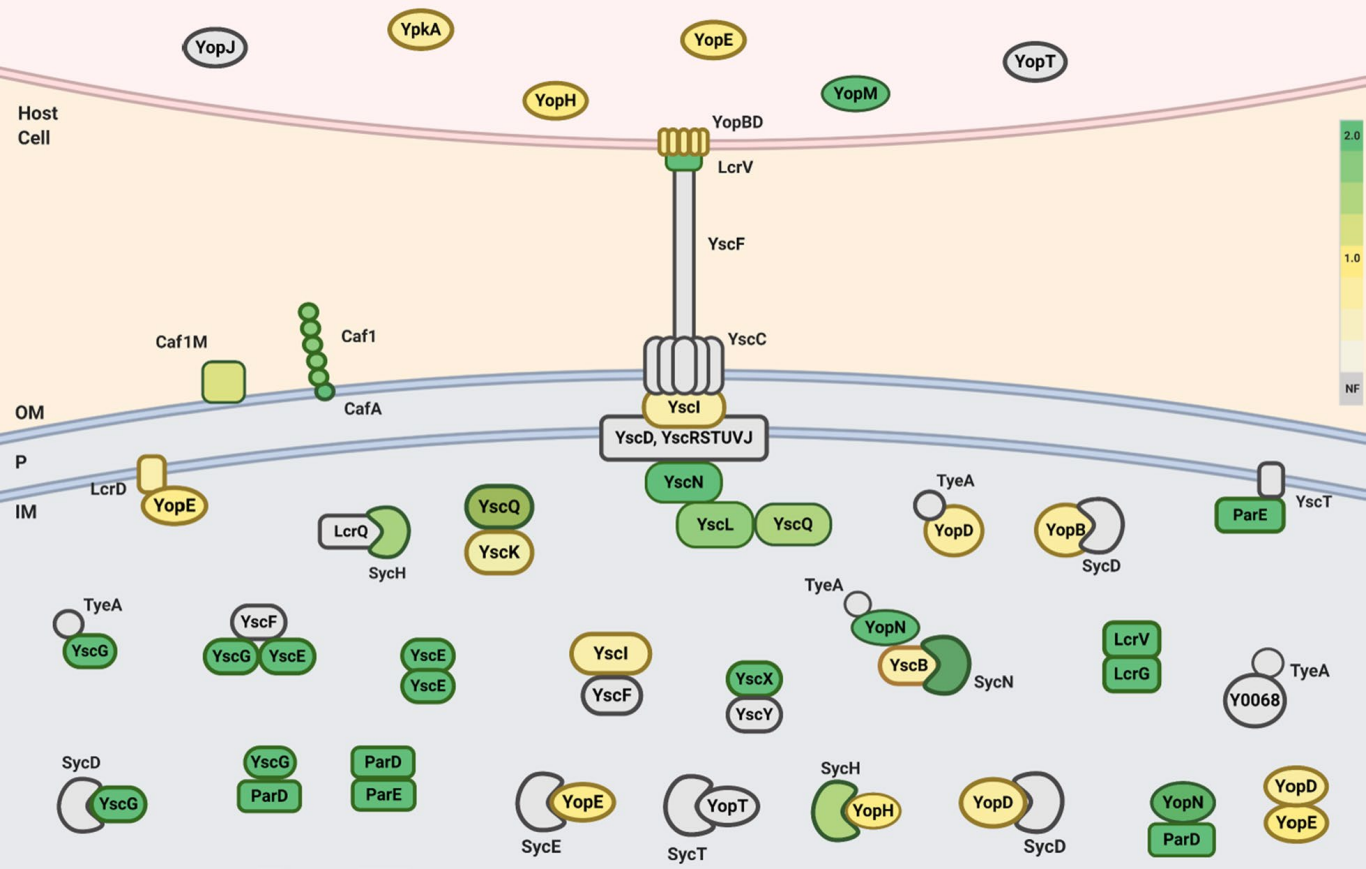
T3SS protein enrichment on Nanotraps. Illustrated model of *Yersinia pestis* type III secretion system (T3SS) with regulatory virulence factors colored according to enrichment value (green ≥ 2, yellow < 2, grey = not found by Nanotraps

The *Y. pestis* T3SS is comprised of over 20 proteins, each capable of forming both stable and transient interactions with other *Yersinia* and host proteins [33]. We have found multiple of these components at higher rates in Nanotrap (CN3080) samples (Fig. 8). Identifying these T3SS proteins and other virulence factors provides us the opportunity to use these as potential future diagnostic markers.

Low calcium response (Lcr) proteins LcrD [33], LcrG [34, 35], LcrH [36, 37], and LcrV were found prominently within Nanotrap samples in both 28 °C and 37 °C grown WCL samples. Notably LcrG and LcrV were found in many Nanotrap samples, and LcrG was enriched from samples grown at 37 °C with the use of Nanotraps. T3SS chaperone protein LcrG acts as a regulator of LcrV secretion and exists as both a cytoplasmic and secreted protein. Given the known binding of LcrV and LcrG and their elevated enrichment scores in some sample types may suggest Nanotrap binding of the LcrGV complex.

Four of the six translocated Yop effectors (YopE, YopH, YpkA, and YopM) [38] were found associated with the CN3080 Nanotraps, although were not found to be enriched. Other Yop proteins such as YopB, YopD, YopK, and YopN were also found within Nanotrap samples. YopB and YopD effectors are outer pore-forming proteins acting in conjunction with LcrV to form the translocon [39]. Though YopB and YopD were present in Nanotraps samples, they were not found to be significantly enriched.

Additionally, several interacting pairs of proteins were found to both be enriched, such as putative toxin-antitoxin proteins ParD and Y0075 (ParE). Our data support higher enrichment scores for some chaperone proteins pulled from 37 °C cell lysate (Additional file 1: Figure S6); for example, we found T3SS chaperones SycH and SycE, while others were not found within our data set. We identified structural components of the injectosome such as YopBD, LcrV, YscI, YscL, and YscQ associated with Nanotraps; however, we were unable to find either of the intermembrane rings or the YscF needle. We found high (> 2) enrichment scores for YscN, YscL, and LcrV. In this work, LcrV has been shown to be enriched by CN3080 Nanotraps through both mass spectrometry and western blotting, especially in unlysed cells and supernatant.

## Discussion

Here, we characterized the ability of Nanotrap particles (type CN3080) to bind *Yersinia pestis* bacteria, proteins, and nucleic acids. We found that the bacteria associate tightly with the Nanotraps, even after washing. We were able to observe the interaction of the bacteria with the Nanotraps by electron microscopy. Experiments showed enhanced nucleic acid yield with the use of Nanotraps. CN3080 Nanotraps effectively concentrated many *Yersinia* proteins for MS detection and allowed visualization of LcrV from blood samples. The magnetic property of these particles made the process more rapid and facilitated the workflow. The large, planar dye molecule that is the affinity bait has relatively non-specific binding properties, and thus can sample a wide variety of biomarkers, which can enhance the detection of low abundance biomarkers [14–16]. It is not known what functional moieties on the bacterial surface participate in this interaction with the Nanotraps, although some outer membrane proteins were identified as binding the Nanotraps.

As plating is the gold standard for *Yersinia pestis* diagnosis, we sought to determine the affinity that CN3080 Nanotraps have for intact bacteria in whole human blood. We found that the Nanotraps have the capacity to bind to a significant number of *Yersinia pestis* bacteria, and that this binding was stable with repeated washings. However, the Nanotraps are unable to bind to all of the bacteria in the sample and cannot “clear” the sample of bacteria, so they are useful for downstream processing but not for quantitative measure or removal of *Yersinia* bacteria.

The circumstances under which free *Yersinia pestis* nucleic acid might be present in blood or plasma samples may be as a result of bacterial cell death and release of their nucleic acid through other mechanisms. The CN3080 Nanotraps were shown to be quite effective at binding *Yersinia* nucleic acids in biological matrices such as whole human blood. Nanotraps might be useful to enrich nucleic acids in upstream steps of a workflow that involves complex biological matrices such as clinical samples. Because secondary verification is necessary for appropriate diagnosis, the use of CN3080 Nanotraps may prove beneficial for early detection and help expedite diagnosis.

While the binding capacity of the particles is not highly specific (rather it provides a “sampling” of the biomarkers present) and can be affected by the biological matrix (whole blood vs. plasma vs. PBS, for example), we found overall that a wide range of *Yersinia pestis* proteins could be found associated with the Nanotrap CN3080 particles. Due to the nature of the Nanotrap dye baits, this interaction is likely to be relatively non-specific, representing the sum of interaction between the bacteria and the magnetic and hydrogel material of the particle as well as with the dye-bait enclosed within the particle. This generally “non-specific” binding can be an advantage to using the Nanotrap particles, as it does not preclude broader binding capacity compared to an antibody-decorated particle for example, and allows wide “sampling” of the proteins in the sample. Different Nanotrap formulations may bind a different profile of proteins, thus broadening the sampling of any particular sample [15, 16, 40].

We found that the Nanotrap particles enhance the mass-spectrometry based detection of *Yersinia* proteins, including the well-known protein antigens F1 and LcrV, as well as PsaA. Nanotraps may be useful as an additional research tool for investigational purposes, as shown by the ability to concentrate secreted proteins and “clean up” a protein sample from whole blood prior to western blotting.

The *Yersinia pestis* V antigen (LcrV) is a multifunctional virulence factor, responsible for regulating the translocation of its cytotoxic effector proteins into host eukaryotic cells through a type III secretion system (T3SS). Due to the protein’s immunosuppressive qualities and effective neutralization through antibody treatment, LcrV has been a target for vaccine development [28, 29, 41]. The Fraction 1 antigen (F1) acts as an antiphagocytic barrier expressed on the outside of the bacterium during mammalian infection [42]. These F1 polymers are released from bacterial cells and shed into the system of the host, acting as a definitive marker of *Y. pestis* infection. F1 has been shown to give anti-plague immunity in animal models against pneumonic and bubonic forms [42]. The identification of LcrV and F1 bound to Nanotraps suggests that Nanotraps may be a useful upstream enhancement to a workflow that may detect these antigens such as lateral flow assays, or other protein based detection methods. The F1 and LcrV antigens are currently used as a diagnostic marker in dipstick, lateral flow and other clinical approaches [43–45]. Thus, Nanotraps could potentially enhance the sensitivity of such diagnostic tools. In addition, the Nanotraps can enhance detection of low-abundance proteins such as secreted proteins.

We characterized the ability of CN3080 Nanotrap particles to bind *Yersinia pestis* intact bacteria, proteins, as well as nucleic acids. Nanotraps may thus provide a useful new upstream sample-processing step to enhance the detection and diagnosis of *Yersinia pestis* in human plasma and whole blood, which are clinically relevant samples. This approach can enhance molecular assays such as PCR-based methods or protein-based methods such as Western Blotting. The integration of Nanotraps into a blood collection device could provide a useful technical improvement of the ability to bind and detect these *Yersinia* analytes.

## Supplementary Information

**Additional file 1:** Additional Figure S1 to S8.

**Additional file 2: Table S1.** Mass spectrometry data from whole cell lysate of *Yersinia pestis* grown on BHI agar at 28 °C bound to Nanotraps (CN3080s), Sample 1. * = not found in control sample; Grey = the #PSMs do not reach enrichment calculation threshold.

**Additional file 3: Table S2.** Mass spectrometry data from whole cell lysate of *Yersinia pestis* grown on BHI agar at 37 °C bound to Nanotraps (CN3080s), Sample 2. * = not found in control sample; Grey = the #PSMs do not reach enrichment calculation threshold**.**

**Additional file 4: Table S3.** Mass spectrometry data from whole cell lysate of *Yersinia pestis* grown in BHI broth at 37 °C bound to Nanotraps (CN3080s), Sample 3. *not found in control sample; Grey = the #PSMs do not reach enrichment calculation threshold.

**Additional file 5: Table S4.** Mass spectrometry data from whole cell lysate of *Yersinia pestis* grown in BHI broth at 37 °C bound to Nanotraps (CN3080s) and washed with DPBS, Sample 4. *not found in control sample; Grey = the #PSMs do not rea enrichment calculation threshold.

**Additional file 6: Table S5.** Mass spectrometry data from intact, whole *Yersinia pestis* bacteria grown in BHI at 37 °C bound to Nanotraps (CN3080s), Sample 5. * = not found in control sample; Grey = the #PSMs do not reach enrichment calculation threshold.

**Additional file 7: Table S6.** Mass spectrometry data from cell supernatant (secreted proteins) of *Yersinia pestis* grown in BHI at 37 °C bound to Nanotraps (CN3080s), Sample 6. * = not found in control sample.

**Additional file 8: Table S7.** Panther Overrepresentation Test using proteins with greater than 2.0 enrichment score from sample W37A. Sheet 1: Biological processes, Sheet 2: Molecular function, Sheet 3: Cellular component.

## Data Availability

All data and materials are available within the manuscript and in additional files.
