# Supplementary material for: Use of magnetic nanotrap particles in capturing Yersinia pestis virulence factors, nucleic acids and bacteria
Source: J Nanobiotechnology. 2021 Jun 21;19:186. doi: 10.1186/s12951-021-00859-8 (PMC8215484; doi:10.1186/s12951-021-00859-8)
Supplement: Supplementary file 1 — Additional file 1: Additional Figure S1 to S8. [file 12951_2021_859_MOESM1_ESM.pdf]

Supplemental Figures and Legends

Figure S1. Effect of different concentrations of Nanotrap on *Yersinia* viability. 10^6 CFU *Y. pestis* bacteria were plated with 0-100uL/mL concentrations of Nanotraps on agar plates and enumerated. Samples were plated directly and did not undergo the binding protocol. No significant difference was found between concentrations of CN3080s suggesting no direct toxicity of the Nanotraps.

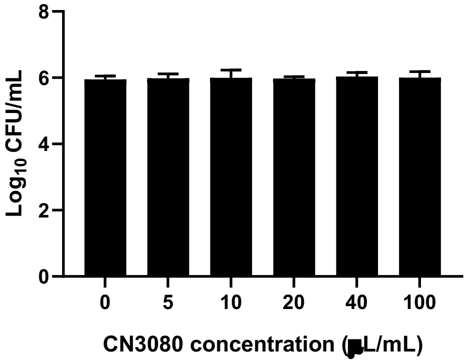

Figure S2. Binding to CN3080 Nanotraps and Baseline Stability of *Yersinia* in whole human blood. 10^6 CFU *Y. pestis* bacteria were spiked into whole human blood and incubated with mixing at the indicated temperature for 0, 24, 48, 72, or 96 hours and processed by binding (bars) or stability (dashes) protocol. (A) Room temperature, (B) 37°C, (C) 40°C, (D) 54°C

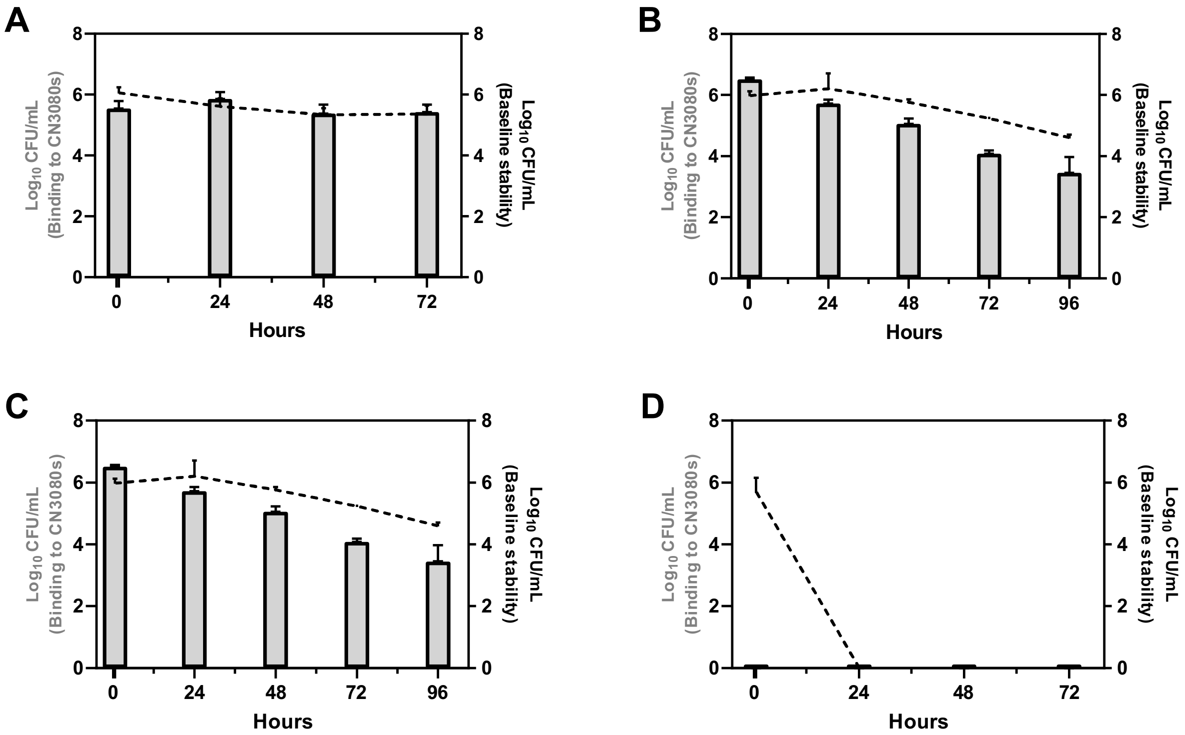

Figure S3. Temperature-dependent cell death of *Yersinia* with and without Nanotraps (A) *Yersinia pestis* survival following incubation at 54°C with and without CN3080 Nanotraps. Bacteria were undetectable from Nanotrap samples (light grey= liquid Nanotraps, dark grey= lyophilized Nanotraps) by the 60m incubation time point, while samples without Nanotraps (dashed line) were capable of surviving until 80m. Results suggest that Nanotraps may slightly promote cell death at elevated temperatures. (B) Comparison of stability and binding protocols over 96-hour incubation at 37°C. Again, *Yersinia* bacteria without Nanotrap survived at significantly higher levels that those incubated with Nanotrap.

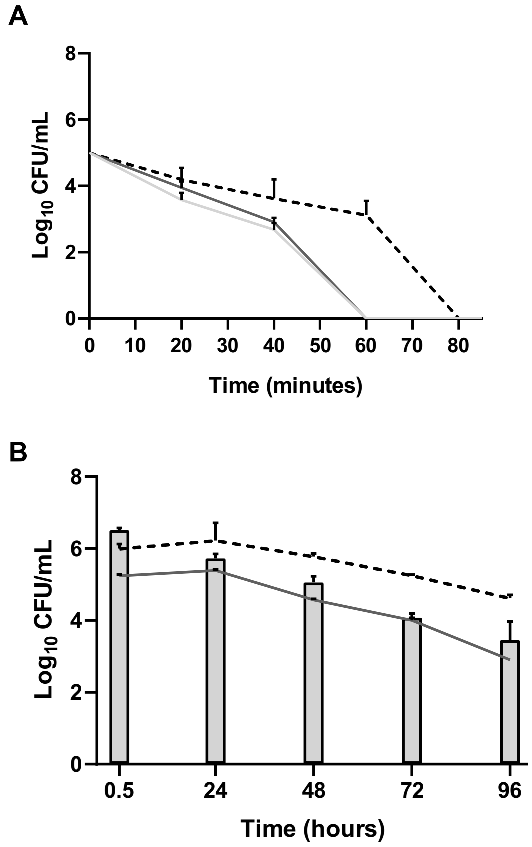

Figure S4. *Yersinia pestis* gDNA binding following 24 hour incubation at the noted temperature. A. Visualization by agarose gel showed Nanotraps were capable of binding *Yersinia pestis* nucleic acid at elevated temperatures. B. Quantitation of the bands showed no significant difference in the amount of gDNA present when bound to Nanotrap or not. The total amount of gDNA decreased with increasing temperature.

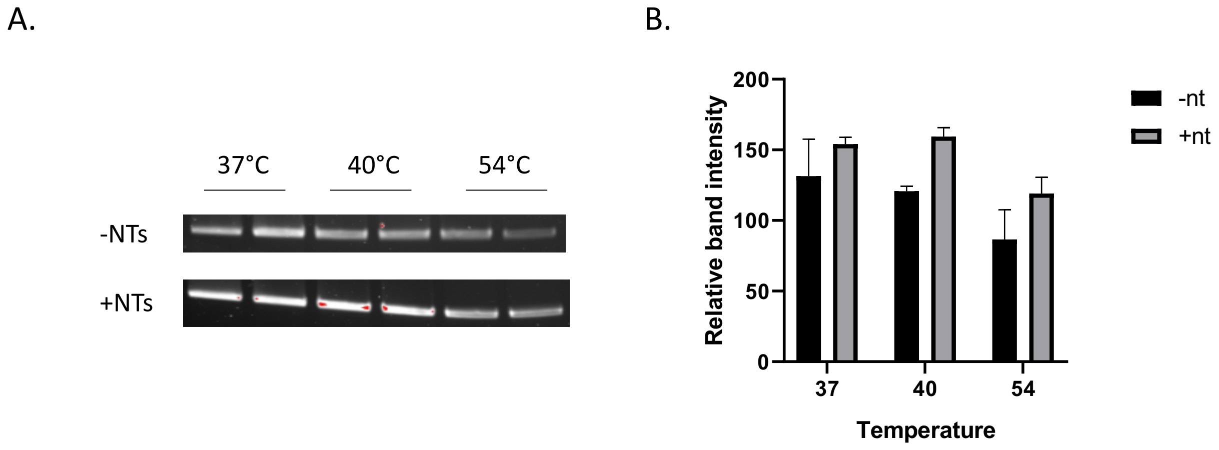

Figure S5. Western blot probing for F1 antigen from *Yersinia* culture supernatant (secreted proteins). The addition of CN3080 Nanotraps did not result in increased band density, suggesting low binding affinity between this variety of Nanotraps and the *Yersinia* F1 antigen.

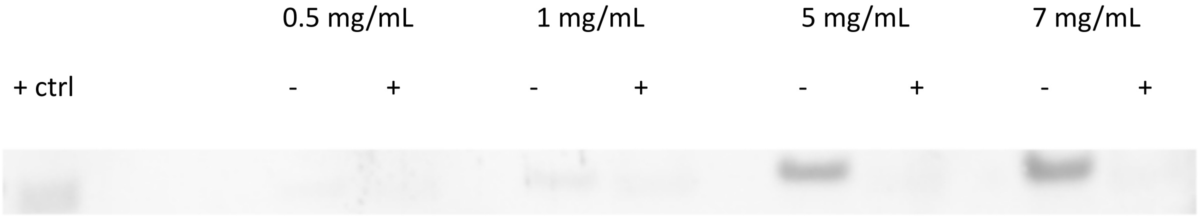

Figure S6. Box and whisker plot of protein enrichment scores organized by selected Gene Ontology groups following Nanotrap binding. Protein stabilization (GO:0050821) proteins showed consistently elevated enrichment scores, while metal ion binding groups showed no definitive patterns. Results suggest that Nanotrap binding to individual proteins may not correspond to protein function or grouping.

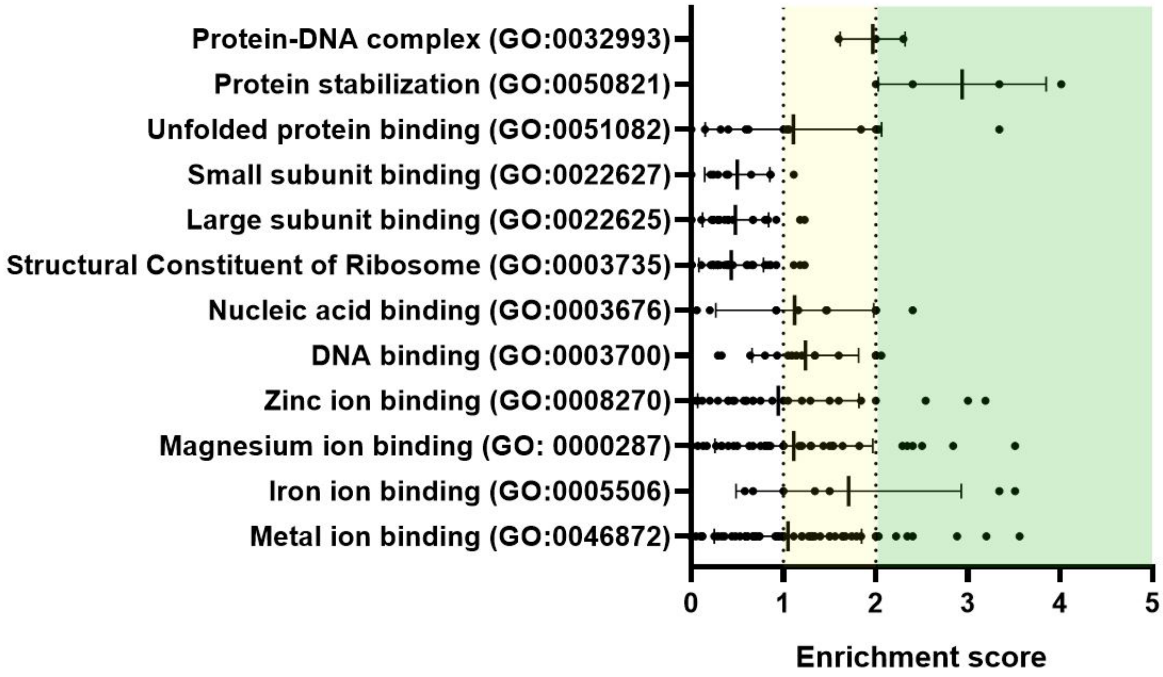

Figure S7: The collision-induced dissociation (CID) spectra for identification of protein LcrV by LC-MS/MS. (a) CID spectrum of the identified peptide FNSAIEALNR (2+ ion with m/z 567.798) of LcrV; (b) CID spectrum of the identified peptide HLSSSGTINIHDK (3+ ion with m/z 470.244) of LcrV. The top panel is the table of the fragment assignments of the peptide, in which the matched b ions are colored with red and y ions with blue. The spectrum (lower panel) is labeled to show b ions, y ions, and neutral loss of water and amine from parent ions.

(a)

#1	b^+	b^{2+}	Seq.	y^+	y^{2+}	#2
1	148.07569	74.54148	F			10
2	262.11862	131.56295	N	987.52178	494.26453	9
3	349.15065	175.07896	S	873.47886	437.24307	8
4	420.18776	210.59752	A	786.44683	393.72705	7
5	533.27182	267.13955	I	715.40971	358.20850	6
6	662.31442	331.66085	E	602.32565	301.66646	5
7	733.35153	367.17940	A	473.28306	237.14517	4
8	846.43559	423.72144	L	402.24594	201.62661	3
9	960.47852	480.74290	N	289.16188	145.08458	2
10			R	175.11895	88.06311	1

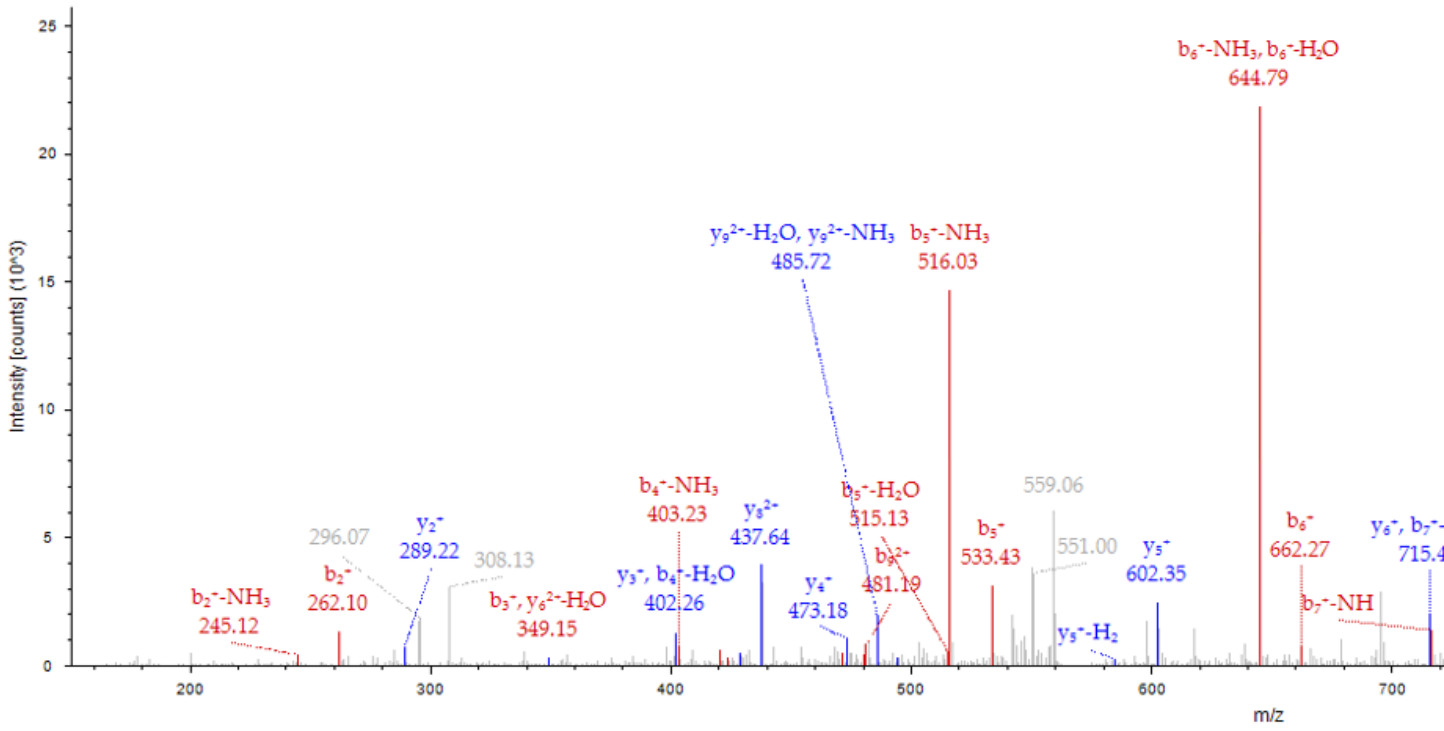

(b)

#1	b⁺	b²⁺	b³⁺	Seq.	y⁺	y²⁺	y³⁺	#2
1	138.06619	69.53673	46.69358	H				13
2	251.15025	126.07876	84.38827	L	1271.65901	636.33314	424.55785	12
3	338.18228	169.59478	113.39894	S	1158.57494	579.79111	386.86316	11
4	425.21431	213.11079	142.40962	S	1071.54291	536.27509	357.85249	10
5	512.24634	256.62681	171.42030	S	984.51088	492.75908	328.84181	9
6	569.26780	285.13754	190.42745	G	897.47886	449.24307	299.83114	8
7	670.31548	335.66138	224.11001	T	840.45739	420.73233	280.82398	7
8	783.39954	392.20341	261.80470	I	739.40971	370.20850	247.14142	6
9	897.44247	449.22487	299.81901	N	626.32565	313.66646	209.44673	5
10	1010.52653	505.76691	337.51370	I	512.28272	256.64500	171.43243	4
11	1147.58545	574.29636	383.20000	H	399.19866	200.10297	133.73774	3
12	1262.61239	631.80983	421.54231	D	262.13975	131.57351	88.05143	2
13				K	147.11280	74.06004	49.70912	1

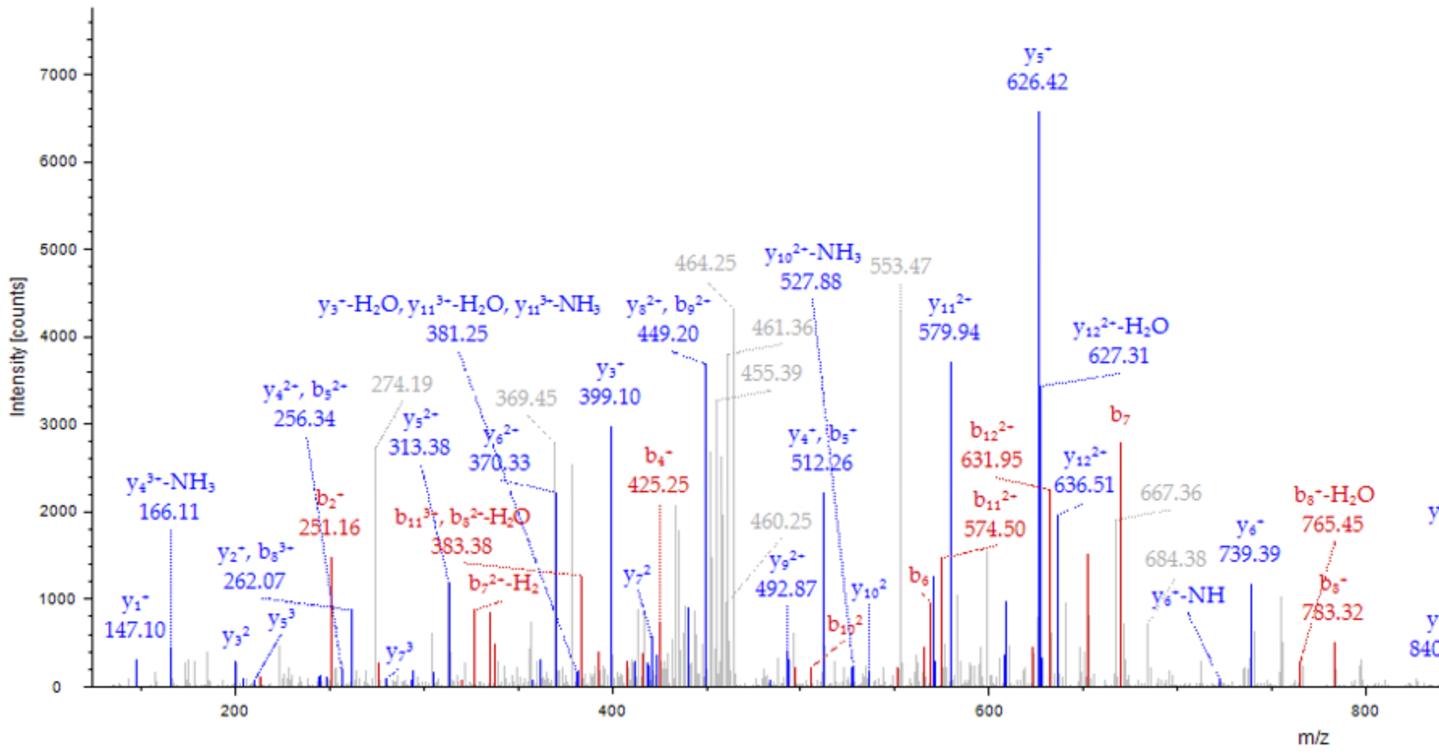

Figure S8: Further analysis of interaction of Reactive Red 120 with *Y. pestis* proteins PsaA and LcrV. The active sites of PsaA and LcrV were highlighted; one for galactose-binding site (PsaA)[31] and the other for TLR2 interaction region (LcrV)[32]. The strongest binding sites of Reactive Red 120 were exactly correlated to these active sites. **A.** PsaA and Galactose-binding site [31]. **B.** LcrV and TLR2 interaction region[32]. The major interactions, hydrogen bonds, between active sites and Reactive Red 120 were labeled with distances of H-bonds which were plotted next to the highlighted graph. **C, D, E**. LcrV-Reactive Red 120 Protein-Dye Interactions up close.

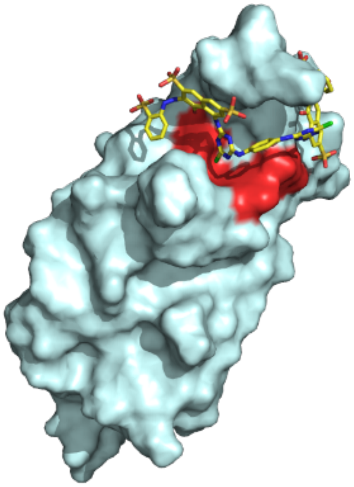

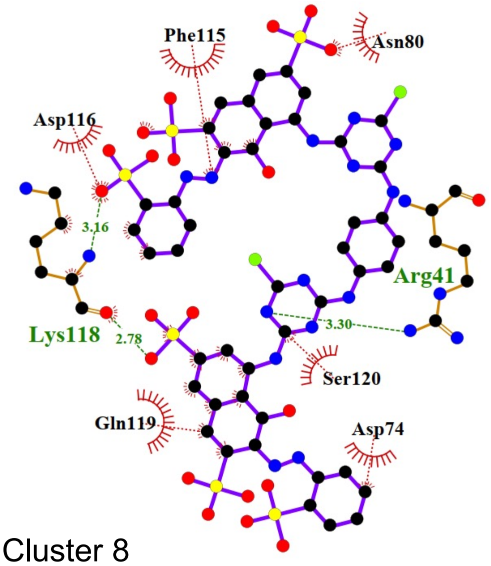

Cluster 8

Cluster serial	Estimated ∆G (Kcal/mol)
8	-10.01
6	-9.6
2	-9.54
11	-9.45
0	-9.44

A.

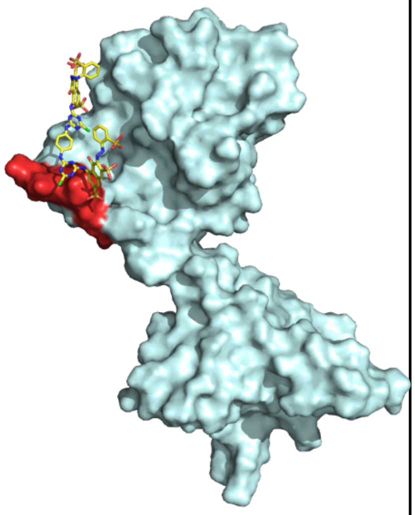
Cluster 4

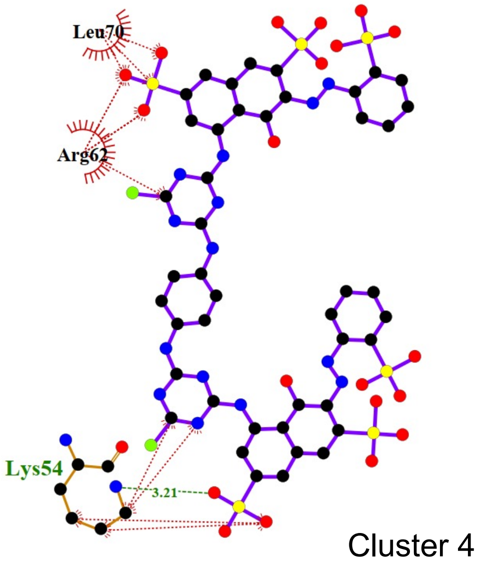

Cluster 4

Cluster serial	Estimated ΔG (Kcal/mol)
4 (36)	-11.59
19 (152)	-10.75
31 (255)	-10.49
18 (144)	-10.48
0 (4)	-10.25

B.

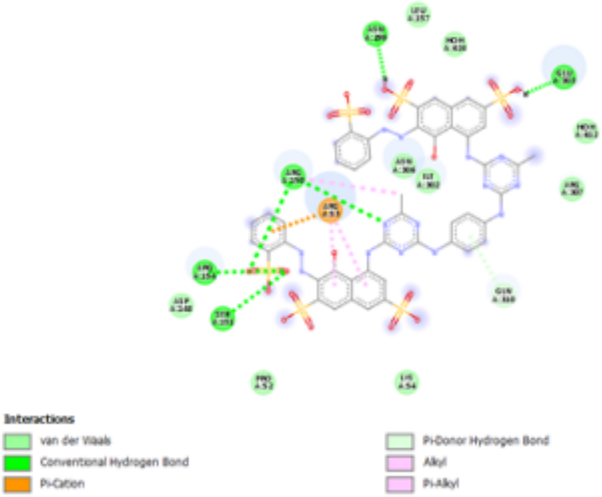

C.

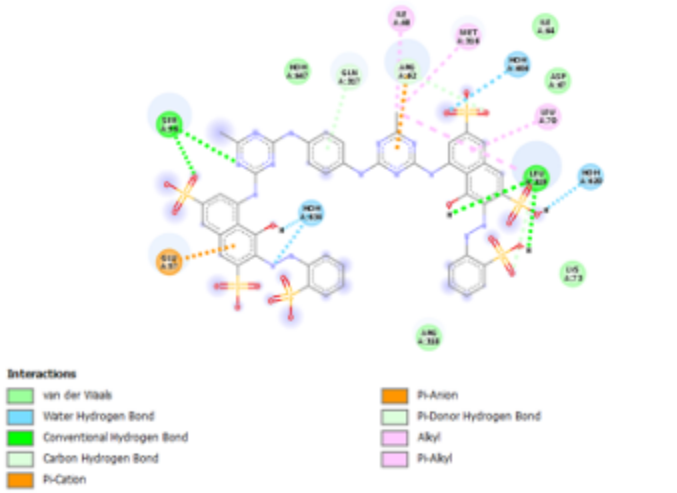
Interactions
van der Waals
Water Hydrogen Bond
Conventional Hydrogen Bond
Carbon Hydrogen Bond
Pi-Cation
Pi-Anion
Pi-Donor Hydrogen Bond
Alkyl
Pi-Alkyl

D.

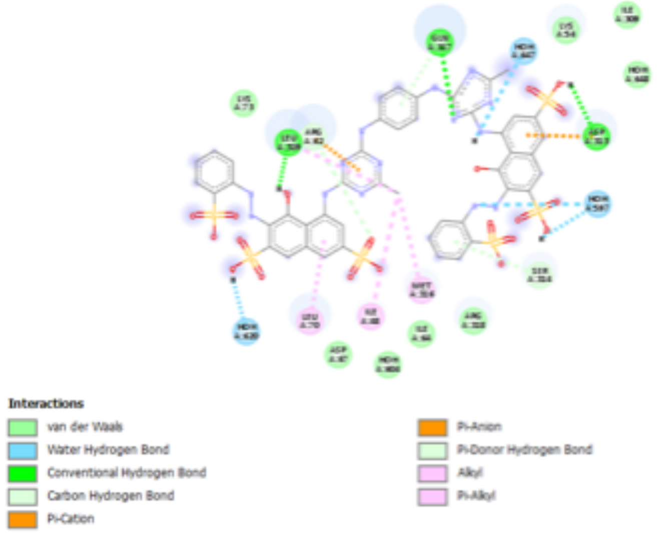
Interactions
van der Waals
Water Hydrogen Bond
Conventional Hydrogen Bond
Carbon Hydrogen Bond
Pi-Cation
Pi-Anion
Pi-Donor Hydrogen Bond
Alkyl
Pi-Alkyl

E.
